# Size and Ecology of a Giant *Pavona clavus* Coral Colony in the Kingdom of Tonga

**DOI:** 10.1002/ece3.73940

**Published:** 2026-07-01

**Authors:** Samantha Crisp, Dennis van Hulten, Lucy Southworth, Tevita Havea, Viliami Fatongiatau, Martin Finau, Tom C. L. Bridge, Patrick Smallhorn‐West

**Affiliations:** ^1^ College of Science and Engineering James Cook University Townsville Queensland Australia; ^2^ School of Biological Sciences University of Auckland Auckland New Zealand; ^3^ California Academy of Sciences San Francisco California USA; ^4^ Ministry of Fisheries Tongatapu Tonga; ^5^ Biodiversity and Geosciences Program Queensland Museum Tropics Townsville Queensland Australia

## Abstract

Some individual coral colonies can grow to immense sizes and survive for centuries, offering rare insight into how corals persist over long time scales. This study documents one of the largest coral colonies ever recorded—a colossal *Pavona clavus* in Tonga—and shows that, despite being dominated by a single species, it supports fish communities and ecosystem functions comparable to typical nearby reefs. These findings suggest that giant coral colonies may play an important role as resilient habitats and potential refuges in a changing ocean.

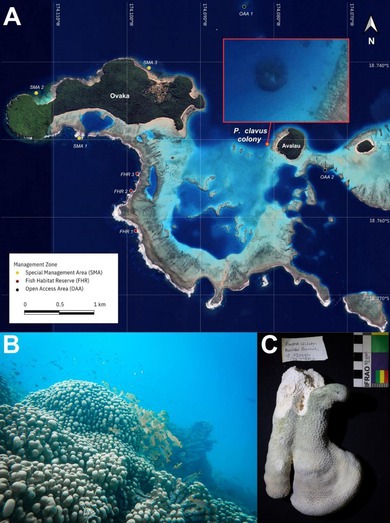

Coral reef ecosystems are generally considered to be diverse, dynamic, and heterogeneous environments (Connell 1978; Fisher et al. 2015; Hatcher 1997). However, in stable conditions, some massive coral colonies can grow to extraordinary sizes (i.e., > 180 m in circumference National Geographic Society 2024) and persist for hundreds, if not thousands of years (i.e., > 500 years Coward et al. 2020; Soong et al. 1999; Takeuchi and Yamashiro 2017). At a time when coral reef degradation is increasingly documented at a global scale (Hughes et al. 2018; Pandolfi et al. 2003; Rocha et al. 2018), there is growing urgency to record the occurrence and ecology of these giant phenomena.

Whilst conducting annual ecological monitoring in affiliation with the Tongan Ministry of Fisheries, a previously unrecorded giant *Pavona clavus* colony—so large it is visible on satellite imagery—was observed in the Ovaka region of the Vava'u island group, Kingdom of Tonga (Figure 1). The coral colony described in this study is located on the sheltered, sandy fore‐reef of Avalau Island (18.75055° S, 174.0809° W), approximately 70 m from the island's nearest fringing reef. It is situated on a gentle slope, with depths ranging from 10 m (shallow edge) to 14 m (deep edge), and its crest rising to approximately 4 m below the surface. This coral colony is well‐known to the Ovaka community, and fisheries access to the colony is restricted to community residents under a local “Special Management Area.”

**FIGURE 1:**
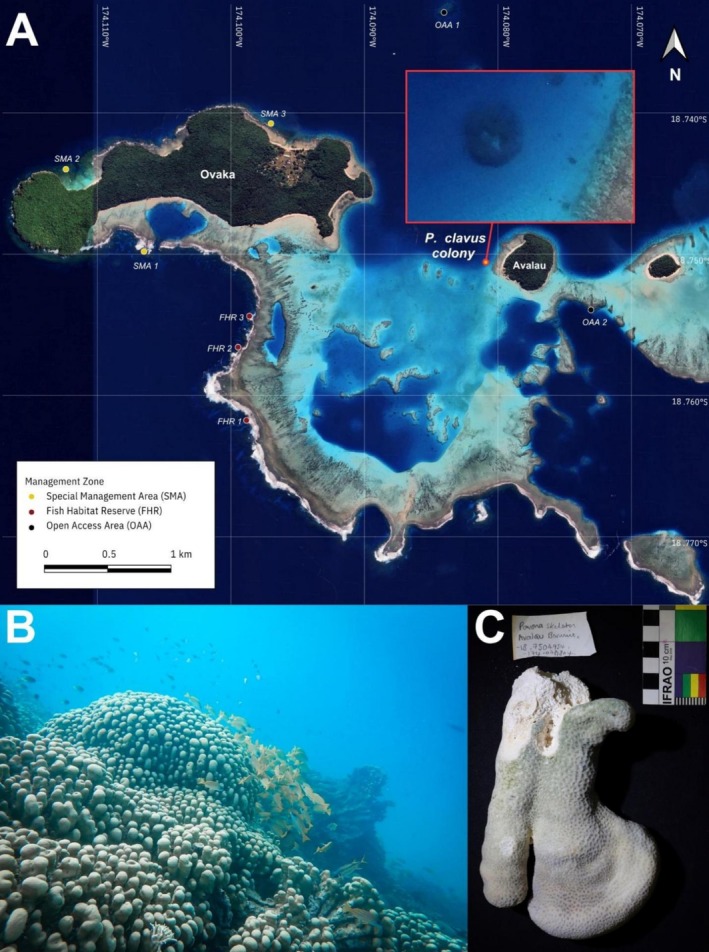
(A) Satellite imagery of the ecological monitoring sites in the Ovaka region of the Vava'u island group, Kingdom of Tonga. The giant *Pavona clavus* colony is located near Avalau Island (18.7506° S, 174.0809° W), and the other surveyed sites are distributed throughout different fisheries management zones of the Ovaka community “Special Management Area.” (B) In situ image of the colony. (C) High resolution image of the skeleton. NB: This figure was constructed using QGIS (QGIS Development Team 2025) and Google Satellite Imagery (Google 2025).

Underwater surveys of the colony were conducted on scuba on 28 October 2024 (Figure 1). This involved three‐dimensional photogrammetry to calculate specific colony metrics, and underwater visual census of fishes to estimate the fish density, biomass, productivity, and species richness associated with the colony (see Supplementary Methods for detailed methods). These methods aligned with the ecological monitoring procedures employed across the Ovaka region to ensure comparability. A fragment of the colony, approximately 15 cm in length, was also collected and registered in the collection of the Queensland Museum (QM registration number G86099) (Figure 1C). Comparison of gross morphology and corallite size and shape between the voucher specimen and the type specimen of *P. clavus* (Dana 1846) from Fiji (USNM 221) (Figure S1), as well as the relatively close proximity of the study site to the type locality of Fiji, provides high confidence that the species identity is *P. clavus* (Dana 1846). Interestingly, Dana notes in his original description that the species can “cover crowdedly areas of considerable extent.”

Photogrammetry measurements revealed that this giant *P. clavus* colony measures approximately 32 × 28 × 8 m (length × width × height), 135 m in circumference, and 2800 m^3^ in volume (Table 1, Figure 2). Corals of this magnitude have been increasingly documented in recent years, with notable examples including: a *Galaxea* cf. *astreata* colony in Indonesia measuring 58 × 71 × 10 m (length × width × height) and 4000 m^2^ in 2D area (MaRHE Center 2025), and another *P. clavus* colony in the Solomon Islands, with dimensions of 34 × 32 × 5.5 m and 183 m in circumference (National Geographic Society 2024; Table S1). Most recently, the “world's largest coral colony”—measuring 111 m in length and 3971 m^2^ in two‐dimensional area—was reported on the Great Barrier Reef (Australia), and was also identified as *P. clavus* (Hutchins 2026). To date, fewer than 20 exceptionally large (i.e., > ~10 m diameter) coral colonies have been documented globally (Table S1), however, it is likely that many more exist. To ensure the standardization of future research into giant coral colonies, we highlight the importance of using 3D photogrammetry to deliver accurate, high resolution, and reproducible measurements of giant coral colonies, and the documentation of these phenomena on centralized, publicly‐available databases such as “Map the Giants” (MaRHE Center 2025).

**TABLE 1:** Key metrics of the giant *Pavona clavus* coral colony in Vava'u, Tonga. These were calculated from the three‐dimensional model (see Figure 2).

Perimeter (m)	134.9
Diameters (m) at widest point and 90°	Length: 32.0 Width: 28.0
Height (m) at tallest point	7.9
Volume (m^3^)	2800.1
Planar area (m^2^)	724.6
Surface area (m^2^)	4492.7
Footprint‐to‐surface area ratio (2D vs 3D area)	0.62

**FIGURE 2:**
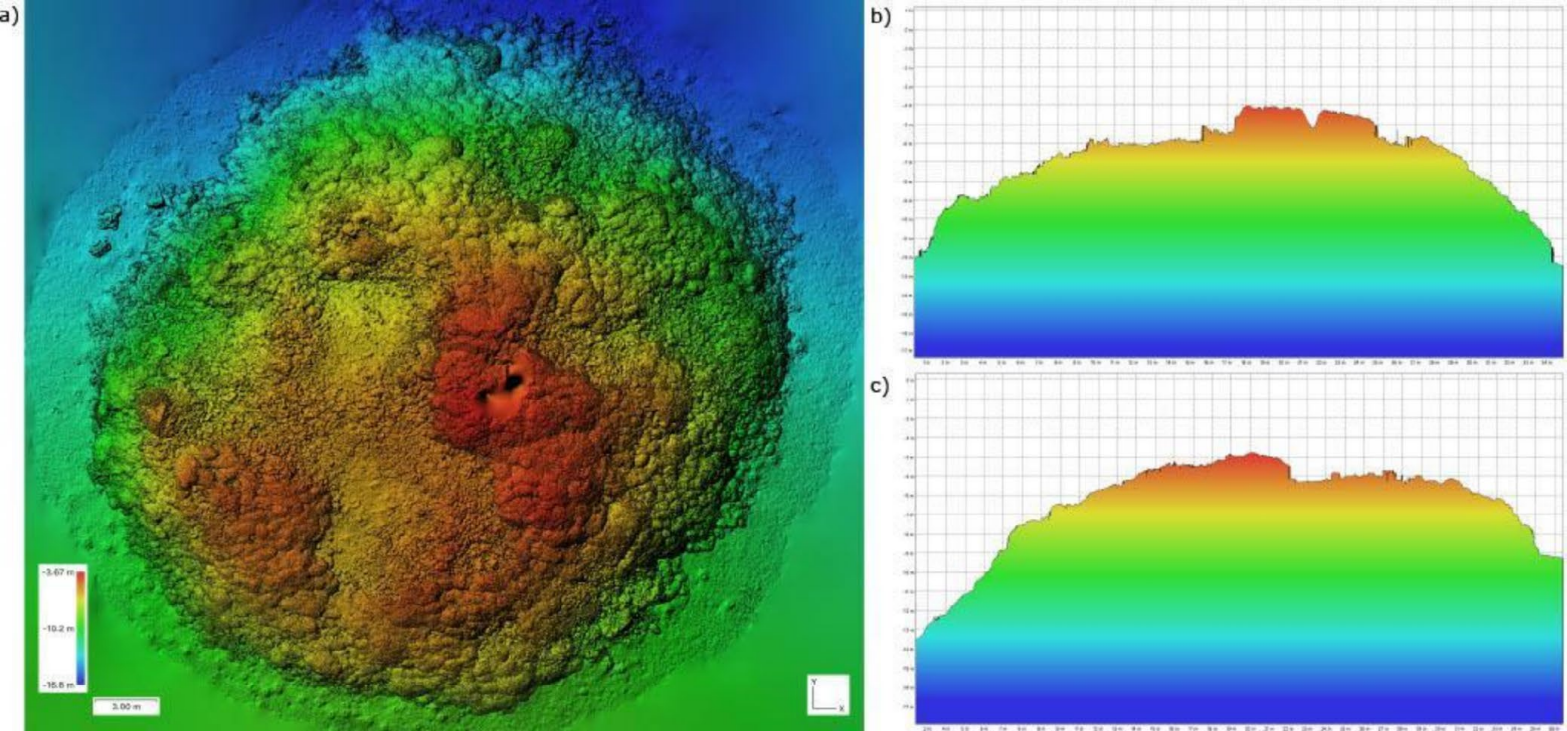
Planar views of the three‐dimensional reconstructed model of the giant Pavona clavus coral colony. Panel (a) depicts the aerial view of the coral colony, and panels (b) and (c) show “length” and “width” cross‐sections of the coral colony topography respectively. The full 3D model of the coral colony can be accessed at: https://sketchfaB.com/3d‐models/crisp‐et‐al‐avalu‐pavona‐bommie‐4280fb30460049b6814322e8b3d849bb.

Beyond its exceptional size, the coral colony documented in this study also supported a remarkably diverse and abundant fish assemblage, which was visually comparable to the fish assemblages of more “typical” (i.e., heterogeneous) reefs in the Ovaka region. Underwater visual census of the fish assemblage associated with the giant coral colony recorded 86 species (Table S2), relative to the average species richness of 85 species/site on neighboring Ovaka reefs. In addition, a total fish count identified a total of 109 reef fish species on the *P. clavus* colony (Table S2), and these belonged to a broad range of trophic groups, including relatively abundant secondary consumers (e.g., piscivores). Further analyses of the fish census data indicated that the colony supported a total fish density of 4.29 ± 0.96SE fish m^−2^, biomass of 1736 ± 61SE kg ha^−1^, and productivity of 2.67 ± 0.88SE kg ha^−1^ day^−1^. Given fishing pressure on the colony is restricted under the Ovaka “Special Management Area,” these values remain within ranges considered relatively high for moderately fished coral reef ecosystems (McClanahan 2018; Smallhorn‐West et al. 2020). These findings are promising regarding the capacity of giant, long‐lived coral colonies to maintain the ecological functions and services expected of more “typical” coral reef systems; however, further research is required to confirm this. In the context of environmental change, understanding the potential for these corals to serve as ecological refugia should also be made a priority.

Looking ahead, substantial knowledge gaps still remain surrounding the ecology and global distribution of giant coral colonies. We suggest that dedicated research into these natural phenomena could provide critical insights into (i) the factors enabling long‐term coral persistence, (ii) the specific ecological roles of giant coral colonies relative to general reef systems, and (iii) how to best conserve these natural phenomena into the future.

## Author Contributions

**Samantha Crisp:** conceptualization (equal), data curation (equal), formal analysis (equal), investigation (equal), methodology (equal), project administration (equal), validation (equal), visualization (equal), writing – original draft (equal). **Dennis van Hulten:** formal analysis (equal), methodology (equal), validation (equal), visualization (equal), writing – review and editing (equal). **Lucy Southworth:** conceptualization (equal), writing – review and editing (equal). **Tevita Havea:** investigation (equal), methodology (equal), writing – review and editing (equal). **Viliami Fatongiatau:** conceptualization (equal), investigation (equal), validation (equal), writing – review and editing (equal). **Martin Finau:** supervision (equal), validation (equal), writing – review and editing (equal). **Tom C. L. Bridge:** supervision (equal), validation (equal), writing – review and editing (equal). **Patrick Smallhorn‐West:** conceptualization (equal), data curation (equal), funding acquisition (equal), investigation (equal), methodology (equal), project administration (equal), resources (equal), supervision (equal), validation (equal), writing – review and editing (equal).

## Funding

Funding provided by National Geographic Society to PSW.

## Conflicts of Interest

The authors declare no conflicts of interest.

## Supporting information


**Figure S1:** Images of a voucher specimen of the colony identified as *P. clavus* (Dana 1846) reported in this study and registered in the collection of the Queensland Museum (G86099) (top), with the type specimen of *P. clavus* (USNM 221) collected from Fiji and housed at the United States National Museum of Natural History—Smithsonian Institution in Washington, DC.
**Table S1:** A catalog of the global occurrence of extraordinarily large coral colonies (i.e., > 10 m diameter). Records of giant coral colonies were sourced from the scientific literature, and from the online platform “Map the Giants” (MaRHE Center 2025). The colonies were then ranked in descending size order, according to their estimated volumes. Volumetric calculations were conducted in ChatGPT (OpenAI 2025), using the generic formula “V = (π*L*W*H)/6” for colonies of half‐ellipsoid morphology. Confidence ranges for the “best” volume estimates (i.e., ±5% uncertainty per dimension, and ±8.66% overall volume) were included, and missing dimensions were inferred from those that were provided (i.e., assuming L = W, W = C/π, H = mean (L,W)/2, and W = 10 for W > 10 records). Potential inaccuracies in volume estimates may result from broad generalizations regarding colony morphology and inconsistent dimension measurements, which highlights the need to use photogrammetry to accurately compare coral colony morphometrics in the future. NB: under “Dimensions (m),” “W,” “L,” “H” and “C” refer to width, length, height and circumference measurements respectively.
**Table S2:** A list of the 109 fish species identified on the giant coral colony, in the Vava'u island group of the Kingdom of Tonga. Species that were identified during a dedicated species count across the entire coral colony area, but not recorded during the underwater visual census (UVC) belt transects, were included in the total species list and indicated by “**.” Subsequent analyses were conducted using the data sourced from the UVC belt transect surveys (i.e., including size and abundance data for 86 reef fish species). Where individuals could not be identified at the species‐level, they were recorded at the genus level (i.e., “Genus” sp.).

## Data Availability

Data is available as a Supporting Information.
