# Supplementary material for: Size and Ecology of a Giant Pavona clavus Coral Colony in the Kingdom of Tonga
Source: Ecol Evol. 2026 Jul 1;16(7):e73940. doi: 10.1002/ece3.73940 (PMC13323178; doi:10.1002/ece3.73940)
Supplement: Supplementary file 1 — Figure S1: Images of a voucher specimen of the colony identified as P. clavus (Dana 1846) reported in this study and registered in the collection of the Queensland Museum (G86099) (top), with the type specimen of P. clavus (USNM 221) collected from Fiji and housed at the United States National Museum of Natural History—Smithsonian Institution in Washington, DC. Table S1: A catalog of the global occurrence of extraordinarily large coral colonies (i.e., > 10 m diameter). Records of giant coral colonies were sourced from the scientific literature, and from the online platform “Map the Giants” (MaRHE Center 2025). The colonies were then ranked in descending size order, according to their estimated volumes. Volumetric calculations were conducted in ChatGPT (OpenAI 2025), using the generic formula “V = (π*L*W*H)/6” for colonies of half‐ellipsoid morphology. Confidence ranges for the “best” volume estimates (i.e., ±5% uncertainty per dimension, and ±8.66% overall volume) were included, and missing dimensions were inferred from those that were provided (i.e., assuming L = W, W = C/π, H = mean (L,W)/2, and W = 10 for W > 10 records). Potential inaccuracies in volume estimates may result from broad generalizations regarding colony morphology and inconsistent dimension measurements, which highlights the need to use photogrammetry to accurately compare coral colony morphometrics in the future. NB: under “Dimensions (m),” “W,” “L,” “H” and “C” refer to width, length, height and circumference measurements respectively. Table S2: A list of the 109 fish species identified on the giant coral colony, in the Vava'u island group of the Kingdom of Tonga. Species that were identified during a dedicated species count across the entire coral colony area, but not recorded during the underwater visual census (UVC) belt transects, were included in the total species list and indicated by “**.” Subsequent analyses were conducted using the data sourced from the UVC belt transect surveys (i.e., i [file ECE3-16-e73940-s001.docx]

**Supplementary Material**

**Title:** Size and ecology of a giant *Pavona clavus* coral colony in the Kingdom of Tonga

Supplementary *Methods*

*Giant coral colony photogrammetry*

Structure from motion photogrammetry was used to reconstruct a three-dimensional model of the *P. clavus* colony. Raw photogrammetry imagery was collected using three GoPro Hero 9 cameras, mounted on a fixed horizontal pole with equal spacing of 90 cm between cameras. Photographs were captured at 1-second intervals, at an average altitude of ~1.5 meter above the coral surface, while maintaining a trajectory parallel to the colony’s topography. Five machine-readable two-dimensional photogrammetry targets (30 cm in length) and one three-dimensional target were placed at points of interest (e.g., the shallowest and deepest areas of the colony) to facilitate correct model orientation and scaling. The survey was conducted in vertical ‘laps’, beginning at the colony base and progressing upward in ~50 cm increments, ensuring complete image coverage of the coral structure.

Photogrammetric processing was performed using Agisoft Metashape Professional v2.1.2 (Agisoft LLC, 2024). Following quality control (i.e. removing poor quality or redundant images), 6,060 images were selected for model reconstruction. Photos were aligned using the high-quality setting, generic preselection, adaptive camera model fitting, a key point limit of 40,000, and a tie point limit of 4,000. This resulted in 6,058 successfully aligned cameras and a sparse cloud consisting of 3,808,557 tie points. The model was scaled and oriented using the machine-readable targets so that the z-axis aligned with depth. After orientation, a Digital Elevation Model (DEM) with a resolution of ~1mm per pixel was constructed using the point cloud as a reference. The mesh was constructed from the depth maps on a high face-count setting, resulting in a high-quality mesh comprising 205,111,482 faces. A small section of the colony that was not imaged during the survey was reconstructed using Metashape’s built-in “close holes” function to ensure the final mesh was watertight. The colony’s 2D area and volumetric measurements were calculated from the DEM, using Metashape’s built-in “measure” tools, restricted to the area containing live coral and excluding adjacent sandy substrate. To estimate surface area, the 3D mesh was analysed using Open3D v0.19.0 (Open3D Contributors, 2025).

*Fish surveys*

Underwater visual census (UVC) of fishes was conducted on scuba, and to ensure comparability, these methods aligned with the monitoring procedures employed at neighboring reefs in the Ovaka region. Fishes were counted and identified to species-level, and their length estimated to the nearest cm. When species could not be accurately identified at the species level (e.g. some juvenile scarids, scallops, etc.), they were classified at the genus level.

With the aim of surveying the entire fish assemblage associated with the *P. clavus* colony, two belt transects encircled the coral colony’s perimeter at depth contours of 13 m (98 m long) and 5 m (65 m long). Each transect employed a two-pass method, involving: 1) a 5 m wide belt transect for all mobile and pelagic fishes, and 2) a 1 m wide belt transect for cryptobenthic and site-attached fish species (Australian Institute of Marine Science (AIMS), 2015; Smallhorn-West, Gordon, et al., 2020). The same two-pass method was employed at neighboring Ovaka reefs, however, at these sites belt transects were standardized to four 30 m transects at each site. To minimize inter-observer variability, all fish surveys were conducted by the same trained observer. The subsequent fish data was then uploaded to the [‘Marine Ecological Research Management Aid’ (MERMAID)](https://explore.datamermaid.org/?lat=-19.916993954009243&lng=-175.01069544554804&zoom=6.560299597818174&country=Tonga&follow_screen=true&benthic_cover=timeSeries&fish_biomass=timeSeries) platform (MERMAID, 2025), to be cleaned, formatted, and integrated with additional ecological data.

In addition to the UVC surveys, a total species count was also estimated for the entire colony area. This was conducted by a separate diver familiar with Indo-Pacific reef fish species and captures the total species assemblage beyond the surveyed transects (Table S2).

*Data analysis*

Four ecological parameters were estimated for the colony’s fish community using the UVC belt transect data, namely: fish density, biomass, productivity, and species richness. Firstly, total fish density (fish m^-2^) was calculated across each transect, as well as species richness (i.e. total number of species) at each respective site. To estimate fish biomass (kg ha^-1^) and productivity (kg ha^-1^ day^-1^) species-level trait data (i.e. maximum terminal length, diet, position in the water column, growth parameters, etc.) were sourced from reference dataframes (Morais & Bellwood, 2018) and the ‘MERMAID’ platform (MERMAID, 2025), before being matched with each species observation. When species-specific data was unavailable, trait data was filled using data for the closest relative (from the literature or FishBase [Froese & Pauly, 2025]), or by the genus average. The latter method was especially useful for the fishes identified at the genus level, and this was calculated using the ‘rfishbase’ package in R (Boettiger et al., 2012) using data extracted from FishBase (Froese & Pauly, 2025). Following the required model parameters, the ‘Otolith’ aging method was assigned to all observations to optimize the accuracy of productivity estimates, and annual mean sea surface temperatures (SSTs) were calculated for the Vava’u region (NOAA Coral Reef Watch, 2025). Once trait data was fully compiled, fish biomass (kg ha^-1^) and productivity (kg ha^-1^ day^-1^) were estimated for each transect replicate, using the ‘rfishprod’ package (Morais & Bellwood, 2018, 2020). These estimates were validated against the biomass estimates calculated on the ‘MERMAID’ platform (MERMAID, 2025).

***Figure S1.*** *Images of a voucher specimen of the colony identified as P. clavus (Dana, 1846) reported in this study and registered in the collection of the Queensland Museum (G86099) (top), with the type specimen of P. clavus (USNM 221) collected from Fiji and housed at the United States National Museum of Natural History – Smithsonian Institution in Washington, DC.*


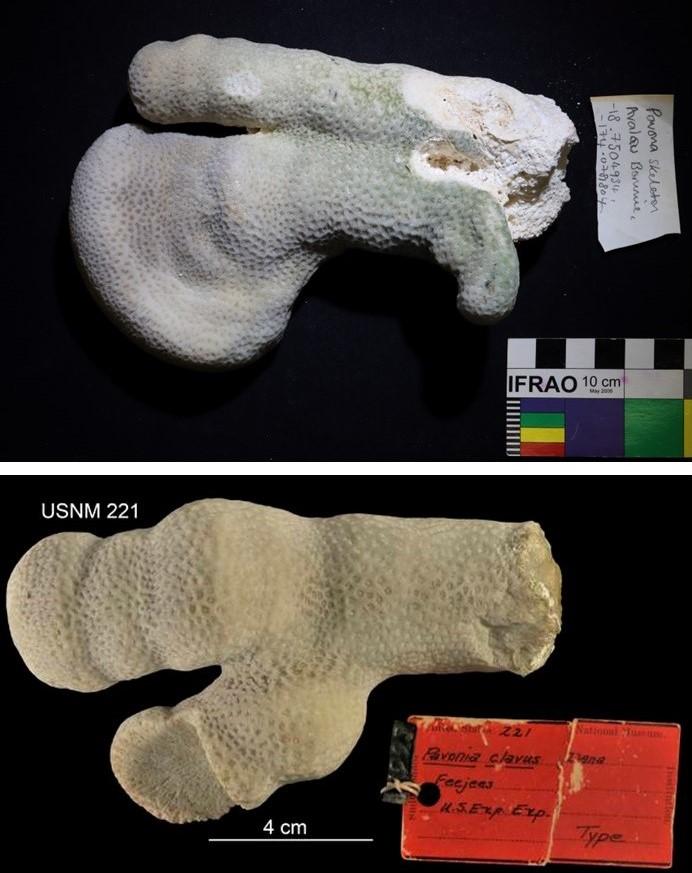


***Table S1.*** *A catalogue of the global occurrence of extraordinarily large coral colonies (i.e. > 10m diameter). Records of giant coral colonies were sourced from the scientific literature, and from the online platform ‘Map the Giants’* (MaRHE Center, 2025)*. The colonies were then ranked in descending size order, according to their estimated volumes. Volumetric calculations were conducted in ChatGPT* (OpenAI, 2025)*, using the generic formula “V = (π*​L*W*H)/6” for colonies of half-ellipsoid morphology. Confidence ranges for the ‘best’ volume estimates (i.e. ±5% uncertainty per dimension, and ±8.66% overall volume) were included, and missing dimensions were inferred from those that were provided (i.e. assuming L = W, W = C/π, H = mean(L,W)/2, and W = 10 for W>10 records). Potential inaccuracies in volume estimates may result from broad generalizations regarding colony morphology and inconsistent dimension measurements, which highlights the need to use photogrammetry to accurately compare coral colony morphometrics in the future. NB: under ‘Dimensions (m)’, ‘W’, ‘L’, ‘H’ and ‘C’ refer to width, length, height and circumference measurements respectively.*

| **Location** | **Year quantified** | **Reference** | **Species** | **Dimensions (m)** | **Age (yrs)** | **Estimated volume range (±8.66% best estimate [m^3^])** |
| --- | --- | --- | --- | --- | --- | --- |
| Australia | 2026 | (Hutchins, 2026) | *Pavona clavus* | L: 111 |  | 94,700 – 112,700  (NB: actual planar area = 3971 m^2^) |
| Indonesia | 2024 | (MaRHE Center, 2025) | *Galaxea astreata* | L: 71  W: 58  H: 10 |  | 19700 - 23400 |
| Solomon  Islands | 2024 | (National Geographic Society, 2024) | *Pavona clavus* | L: 34  W: 32  H: 5.5  C: 183 | 300 - 500 | 2862 - 3405 |
| Tonga | 2025 | *Our study | *Pavona clavus* | L: 32  W: 28  H: 8  C: 135 | 400 - 500 | 2678 - 3170  (NB: actual volume = 2800 m^3^) |
| American Samoa | 2020 | (Coward et al., 2020) | *Porites sp.* | W: 22.4  H: 8  C: 69.2  *3 colonies >10m diameter | 420 - 652 | 2044 - 2432 |
| Maldives | 2024 | (MaRHE Center, 2025) | *Pavona cf. clavus* | L: 20  C: 61 |  | 1843 - 2187 |
| American Samoa | 2009 | (Brown et al., 2009) | *Porites lutea* | L: 17  H: 7  C: 41 | 368 - 571 | 1124 - 1334 |
| Maldives | 2024 | (MaRHE Center, 2025) | *Pavona cf. clavus* | L: 16  W: 16  H: 6.4  C: 54 |  | 981 - 1165 |
| Indonesia | 2025 | (MaRHE Center, 2025) | *Goniopora sp.* | L: 25  W: 8  H: 16 |  | 958 - 1136 |
| Taiwan | 1999 | (Soong et al., 1999) | *Porites lobata* | H: 12  C: 31 | 1000 | 719 - 853 |
| Maldives | 2013 | (MaRHE Center, 2025) | *Turbinaria sp.* | L: 11  W: 6  C: 35 |  | 444 - 529 |
| Japan | 2017 | (Takeuchi & Yamashiro, 2017) | *Porites australiensis* | L: 11.1  C: 33.7 | 500 - 2100 | 329 - 389 |
| Australia | 2021 | (Smith et al., 2021) | *Porites sp.* | W: 10.4  H: 5.3 | 421 - 438 | 273 - 323 |
| Maldives | 2024 | (MaRHE Center, 2025) | *Pavona sp.* | W > 10  H: 4  C: 50 |  | 239 - 284   \|  \| \| --- \|  \|  \| \| --- \| |
| Maldives | 2023 | (MaRHE Center, 2025) | *Porites sp.* | W > 10 |  | 239 - 284 |
| Maldives | 2025 | (MaRHE Center, 2025) | *Pavona cf. clavus* | L: 13  W: 7  C: 35 |  | 228 - 271 |
| Maldives | 2024 | (MaRHE Center, 2025) | *Porites sp.* | L: 11.75  W: 12.1  H: 3.1 |  | 211 - 251 |

***Table S2.*** *A list of the 109 fish species identified on the giant coral colony, in the Vava’u island group of the Kingdom of Tonga. Species that were identified during a dedicated species count across the entire coral colony area, but not recorded during the underwater visual census (UVC) belt transects, were included in the total species list and indicated by ‘**’. Subsequent analyses were conducted using the data sourced from the UVC belt transect surveys (i.e. including size and abundance data for 86 reef fish species). Where individuals could not be identified at the species-level, they were recorded at the genus level (i.e. ‘Genus’ sp.).*

|  | ***Species*** |  |  |
| --- | --- | --- | --- |
| *1* | *Acanthurus lineatus* | *28* | *Chlorurus bleekeri* |
| *2* | *Acanthurus nigrofuscus* | *29* | *Chlorurus spilurus* |
| *3* | *Acanthurus olivaceus* | *30* | *Chromis amboinensis* |
| *4* | *Amblyglyphidodon melanopterus* | *31* | *Chromis atripes* |
| *5* | *Amphiprion chrysopterus* | *32* | *Chromis iomelas* |
| *6* | *Amphiprion melanopus* | *33* | *Chromis lepidolepis* |
| *7* | ***Anampses twistii* | *34* | ***Chromis margaritifer* |
| *8* | *Aphareus furca* | *35* | *Chromis ternatensis* |
| *9* | ***Aprion virescens* | *36* | *Chromis viridis* |
| *10* | *Arothron nigropunctatus* | *37* | ***Chromis weberi* |
| *11* | *Balistapus undulatus* | *38* | *Chromis xanthura* |
| *12* | *Bodianus loxozonus* | *39* | *Chrysiptera talboti* |
| *13* | *Caesio caerulaurea* | *40* | ***Chrysiptera taupou* |
| *14* | *Centropyge bispinosa* | *41* | *Cirrhilabrus punctatus* |
| *15* | *Centropyge flavissima* | *42* | ***Cirripectes stigmaticus* |
| *16* | ***Cephalopholis argus* | *43* | *Ctenochaetus cyanocheilus* |
| *17* | ***Cephalopholis urodeta* | *44* | *Ctenochaetus striatus* |
| *18* | *Chaetodon auriga* | *45* | ***Dascyllus aruanus* |
| *19* | ***Chaetodon lineatus* | *46* | *Dascyllus trimaculatus* |
| *20* | *Chaetodon lunulatus* | *47* | *Epibulus brevis* |
| *21* | ***Chaetodon mertensii* | *48* | *Epibulus insidiator* |
| *22* | *Chaetodon pelewensis* | *49* | *Epinephelus merra* |
| *23* | ***Cheilinus chlorourus* | *50* | *Gnathodentex aureolineatus* |
| *24* | *Cheilinus fasciatus* | *51* | *Gomphosus varius* |
| *25* | *Cheilodipterus artus* | *52* | *Halichoeres melanurus* |
| *26* | ***Cheilodipterus macrodon* | *53* | *Halichoeres prosopeion* |
| *27* | *Cheilodipterus quinquelineatus* | *54* | *Hemigymnus fasciatus* |
| *55* | *Hemigymnus melapterus* | *88* | *Pomacentrus imitator* |
| *56* | *Heniochus chrysostomus* | *89* | *Pomacentrus maafu* |
| *57* | ***Heniochus monoceros* | *90* | *Pomacentrus moluccensis* |
| *58* | ***Koumansetta rainfordi* | *91* | *Pomacentrus vaiuli* |
| *59* | *Labrichthys unilineatus* | *92* | *Priacanthus hamrur* |
| *60* | *Labroides bicolor* | *93* | *Pseudocheilinus hexataenia* |
| *61* | *Labroides dimidiatus* | *94* | ***Pterocaesio trilineata* |
| *62* | *Labropsis australis* | *95* | *Pycnochromis margaritifer* |
| *63* | ***Labrychthys unilineatus* | *96* | *Pygoplites diacanthus* |
| *64* | ***Lenthrinus obsoletus* | *97* | *Sargocentron spiniferum* |
| *65* | *Lutjanus bohar* | *98* | *Saurida gracilis* |
| *66* | ***Lutjanus fulvus* | *99* | ***Scarus altipinnis* |
| *67* | *Lutjanus gibbus* | *100* | *Scarus niger* |
| *68* | *Lutjanus kasmira* | *101* | *Scarus schlegeli* |
| *69* | ***Lutjanus quinquelineatus* | *102* | *Scolopsis bilineata* |
| *70* | *Meiacanthus tongaensis* | *103* | *Stegastes lacrymatus* |
| *71* | *Monotaxis grandoculis* | *104* | *Stegastes nigricans* |
| *72* | *Monotaxis heterodon* | *105* | ***Synodus variegatus* |
| *73* | *Mulloidichthys vanicolensis* | *106* | *Thalassoma hardwicke* |
| *74* | *Myripristis berndti* | *107* | *Thalassoma lunare* |
| *75* | *Myripristis kuntee* | *108* | *Triaenodon obesus* |
| *76* | *Myripristis sp.* | *109* | *Zebrasoma scopas* |
| *77* | *Neoniphon sammara* |  |  |
| *78* | *Oxycheilinus digramma* |  |  |
| *79* | *Oxycheilinus orientalis* |  |  |
| *80* | ***Oxycheilinus unifasciatus* |  |  |
| *81* | *Paracirrhites forsteri* |  |  |
| *82* | *Parupeneus barberinus* |  |  |
| *83* | *Parupeneus cyclostomus* |  |  |
| *84* | *Plagiotremus tapeinosoma* |  |  |
| *85* | *Plectroglyphidodon johnstonianus* |  |  |
| *86* | *Pomacentrus callainus* |  |  |
| *87* | *Pomacentrus coelestis* |  |  |

**References**

Agisoft LLC. (2024). *Agisoft Metashape Professional* (Version 2.1.2) [Computer software]. https://www.agisoft.com

Australian Institute of Marine Science (AIMS). (2015). *AIMS Long-term Monitoring Program: Video and photo transects (Great Barrier Reef)* [Dataset]. https://doi.org/10.25845/5c09bc4ff315c

Boettiger, C., Lang, D. T., & Wainwright, P. C. (2012). rfishbase: Exploring, manipulating and visualizing FishBase data from R. *Journal of Fish Biology*, *81*(6), 2030–2039. https://doi.org/10.1111/j.1095-8649.2012.03464.x

Brown, D. P., Basch, L., Barshis, D., Forsman, Z., Fenner, D., & Goldberg, J. (2009). American Samoa’s island of giants: Massive *Porites* colonies at Ta’u island. *Coral Reefs*, *28*(3), 735. https://doi.org/10.1007/s00338-009-0494-8

Coward, G., Lawrence, A., Ripley, N., Brown, V., Sudek, M., Brown, E., Moffitt, I., Fuiava, B., & Vargas-Ángel, B. (2020). A new record for a massive *Porites* colony at Ta’u Island, American Samoa. *Scientific Reports*, *10*(1), 21359. https://doi.org/10.1038/s41598-020-77776-7

Froese, R., & Pauly, D. (2025). *FishBase* [Dataset]. www.fishbase.org

Hutchins, R. (2026, February 26). *Citizen scientists find the largest known coral on Great Barrier Reef.* Oceanographic. https://oceanographicmagazine.com/news/citizen-scientists-find-largest-known-coral-on-great-barrier-reef/

MaRHE Center. (2025, March 12). *Map the Giants*. Map the Giants. https://www.mapthegiants.com/

MERMAID. (2025). *Marine Ecological Research Management Aid (MERMAID)* [Dataset]. https://datamermaid.org

Morais, R. A., & Bellwood, D. R. (2018). Global drivers of reef fish growth. *Fish and Fisheries*, *19*(5), 874–889. https://doi.org/10.1111/faf.12297

Morais, R. A., & Bellwood, D. R. (2020). Principles for estimating fish productivity on coral reefs. *Coral Reefs*, *39*(5), 1221–1231. https://doi.org/10.1007/s00338-020-01969-9

National Geographic Society. (2024, November 14). *Scientists discover world’s largest coral—So big it can be seen from space*. National Geographic. <https://www.nationalgeographic.com/environment/article/world-largest-coral-colony-discovery>

NOAA Coral Reef Watch. (2025). *Northern Tonga monthly sea-surface temperature time-series* [Dataset]. https://coralreefwatch.noaa.gov/product/vs/data/northern_tonga.txt

OpenAI. (2025). *Half-ellipsoid volume estimates for large coral colonies compiled from published and unpublished sources.* ChatGPT. https://chat.openai.com/

Open3D Contributors. (2025). *Open3D* (Version 0.19.0) [Computer software]. <https://www.open3d.org>

Smallhorn-West, P., Gordon, S., Stone, K., Ceccarelli, D., Malimali, S., Halafihi, T., Wyatt, M., Bridge, T., Pressey, R., & Jones, G. (2020). Biophysical and anthropogenic influences on the status of Tonga’s coral reefs and reef fish fishery. *PLOS ONE*, *15*(11), e0241146. https://doi.org/10.1371/journal.pone.0241146

Smith, A., Cook, N., Cook, K., Brown, R., Woodgett, R., Veron, J., & Saylor, V. (2021). Field measurements of a massive *Porites* coral at Goolboodi (Orpheus Island), Great Barrier Reef. *Scientific Reports*, *11*(1), 15334. https://doi.org/10.1038/s41598-021-94818-w

Soong, K., Chen, C. A., & Chang, J.-C. (1999). A very large poritid colony at Green Island, Taiwan. *Coral Reefs*, *18*(1), 42. https://doi.org/10.1007/s003380050151

Takeuchi, I., & Yamashiro, H. (2017). Large *Porites* microatoll found by aerial survey at Sesoko Island, Okinawa, Japan. *Coral Reefs*, *36*(4), 1317. https://doi.org/10.1007/s00338-017-1626-1
